# A large-scale behavior change intervention to prevent Nipah transmission in Bangladesh: components and costs

**DOI:** 10.1186/s13104-017-2549-1

**Published:** 2017-06-26

**Authors:** Nazmun Nahar, Mohammad Asaduzzaman, Rebeca Sultana, Fernando Garcia, Repon C. Paul, Jaynal Abedin, Hossain M. S. Sazzad, Mahmudur Rahman, Emily S. Gurley, Stephen P. Luby

**Affiliations:** 1 icddr,b, Dhaka, Bangladesh; 2 Swiss Tropical and Public Health Institute, Basel, Switzerland; 3 University of Basel, Basel, Switzerland; 4 FHI360, Washington, D.C., USA; 5 Institute of Epidemiology, Disease Control and Research (IEDCR), Dhaka, Bangladesh; 6 Infectious Diseases and Geographic Medicine, Stanford University, Stanford, CA USA

**Keywords:** Nipah virus infection, Behavior change communication intervention, Prevention, Intervention cost, Bangladesh

## Abstract

**Background:**

Nipah virus infection (NiV) is a bat-borne zoonosis transmitted to humans through consumption of NiV-contaminated raw date palm sap in Bangladesh. The objective of this analysis was to measure the cost of an NiV prevention intervention and estimate the cost of scaling it up to districts where spillover had been identified.

**Methods:**

We implemented a behavior change communication intervention in two districts, testing different approaches to reduce the risk of NiV transmission using community mobilization, interpersonal communication, posters and TV public service announcements on local television during the 2012–2014 sap harvesting seasons. In one district, we implemented a “no raw sap” approach recommending to stop drinking raw date palm sap. In another district, we implemented an “only safe sap” approach, recommending to stop drinking raw date palm sap but offering the option of drinking safe sap. This is sap covered with a barrier, locally called *bana,* to interrupt bats’ access during collection. We conducted surveys among randomly selected respondents two months after the intervention to measure the proportion of people reached. We used an activity-based costing method to calculate the cost of the intervention.

**Results:**

The implementation cost of the “no raw sap” intervention was $30,000 and the “only safe sap” intervention was $55,000. The highest cost was conducting meetings and interpersonal communication efforts. The lowest cost was broadcasting the public service announcements on local TV channels. To scale up a similar intervention in 30 districts where NiV spillover has occurred, would cost between $2.6 and $3.5 million for one season. Placing the posters would cost $96,000 and only broadcasting the public service announcement through local channels in 30 districts would cost $26,000.

**Conclusions:**

Broadcasting a TV public service announcement is a potential low cost option to advance NiV prevention. It could be supplemented with posters and targeted interpersonal communication, in districts with a high risk of NiV spillover.

**Electronic supplementary material:**

The online version of this article (doi:10.1186/s13104-017-2549-1) contains supplementary material, which is available to authorized users.

## Background

Nipah virus (NiV) infection is a fatal emerging zoonosis that can transmit from bats to humans and can cause further person-to-person transmission [1–4]. In Bangladesh, several NiV outbreaks have been identified since 2001, and raw date palm sap consumption has been repeatedly implicated as the pathway of transmission from bats to humans [5, 6]. Raw sap is collected during cold months, from November to March, by shaving the bark near the top of the date palm tree [6, 7]. During sap collection, bats often visit date palm trees and contaminate sap with their saliva and urine [7, 8]. Interrupting bat-to-human transmission may reduce the risk of a potentially large outbreak.

Based on previous pilot studies on interrupting bats access to sap [8–10], and on the Government of Bangladesh’s recommendation to abstain from drinking raw sap, we developed and implemented a behavior change communication intervention using two different approaches to reduce the risk of NiV transmission. After the intervention, local residents’ knowledge of NiV increased, and people reported changing their behavior to reduce the risk of NiV transmission through date palm sap [11]. Thus, understanding the intervention development, process and logistics will help plan scaling it up. Calculating the approximate cost of the intervention, and the proportion of people to be reached, is useful to make investment decisions [12–14] between potential interventions to prevent not just NiV, but other emerging zoonoses.

The objective of our paper is to describe and calculate the cost of an already implemented behavior change communication intervention, and estimate the cost of scaling it up to districts where NiV spillover was identified in Bangladesh, using risk-based scenarios.

## Methods

### Study sites

**Fig. 1:**
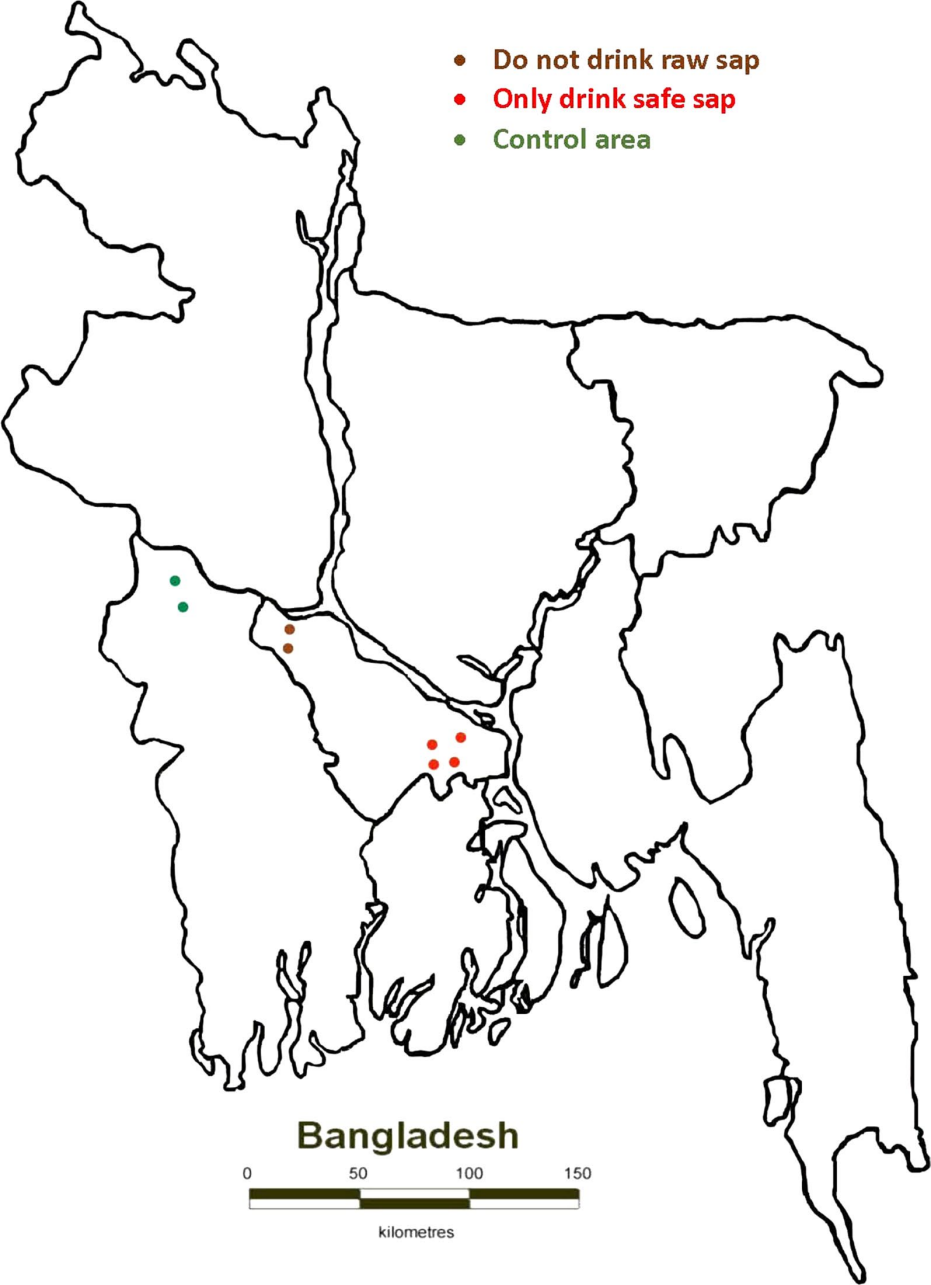
Map of Bangladesh showing the “no raw sap” and the “only safe sap” Nipah prevention intervention areas. Map courtesy Wikimedia Commons, author, CIA, Ananda

We developed a behavior change communication intervention using two separate approaches, targeting rural areas from two NiV endemic districts: Rajbari and Faridpur, where date palm trees are harvested and residents drink raw date palm sap (Fig. 1). We selected these districts because both have been repeatedly affected by NiV outbreaks, both are from the same geographical region, neighboring each other, and have similar raw sap collection and consumption practices. Within those districts, we selected two sub-districts that do not border each other to avoid interference between the interventions. The population of Rajbari and Faridpur study sites was approximately 361,000 and 335,000 respectively.

### Intervention and materials development

**Fig. 2:**
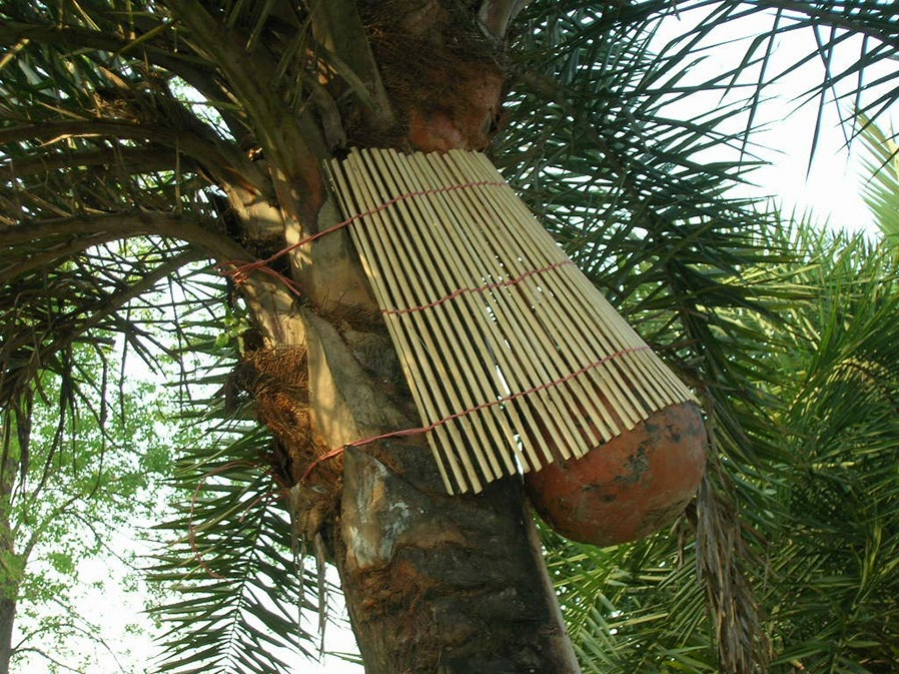
*Bana* to stop bats access to the raw date palm sap to prevent Nipah virus infection in the “only safe sap” area

Following the Government of Bangladesh’s recommendation of abstaining from drinking raw sap, we developed an intervention discouraging people from drinking raw date palm sap in Rajbari District, herein referred to as the “no raw sap” intervention. Some people continued to drink raw sap though they were aware of the risk [15], thus we developed an “only safe sap” intervention in Faridpur District, discouraging drinking raw sap but offering the option of drinking sap protected by a skirt-like barrier locally called *bana* (Fig. 2). During collection, *banas* can stop bats from accessing and contaminating the sap with NiV [8].

We worked with a Bangladeshi communication organization to develop posters, calendars, yearly planners, stickers, sweatshirts and TV public service announcements. Our qualitative research data collection team pre-tested the materials conducting focus group discussions with audiences similar to our target audience. Based on these results, we revised and fine-tuned the messages and illustrations. We also developed training guides for the staff implementing the intervention. The communication organization designed and printed the final training guides.

**Fig. 3:**
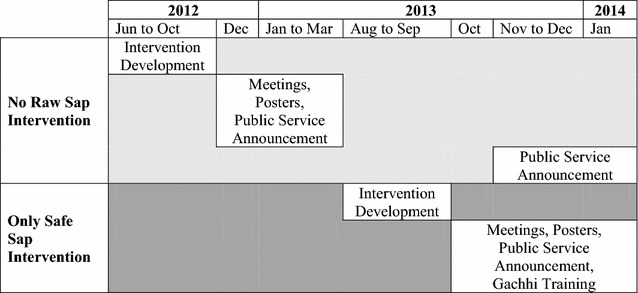
No raw sap” and “Only safe sap” intervention development and implementation to reduce the risk of Nipah virus transmission in Bangladesh during the 2012–2013 and 2013–2014 date palm sap harvesting seasons

We developed the “no raw sap” intervention, including production of the communication materials, from June to October, 2012 and the “only safe sap” intervention from August to September, 2013 (Fig. 3).

### NGO selection

We visited local NGOs from both districts to assess their experience and capability to implement the interventions in the selected sub-districts. Using a competitive bidding process, we selected one local NGO from each district. We assessed their experience with similar interventions, knowledge of the areas to be covered and qualifications of their key personnel. We also compared the size of the organizations, as an indicator of their capacity to implement the intervention, and the budget required to carry it out.

The selected NGOs visited villages and talked to villagers to get an estimate on the number of households, and identify opinion leaders and local sap harvesters (*gachhis).* We provided training to the NGOs’ staff on interpersonal communication, on organizing and conducting meetings with opinion leaders and community residents, and on key intervention messages.

### The intervention implementation

In both intervention areas, the NGOs conducted one opinion leaders and one community meeting per 500 households approximately. Prior to conducting the meetings, the NGOs affixed NiV prevention posters in public places such as health centers, bazaars, and areas with heavy traffic of people. We provided calendars or yearly planners, with NiV prevention messages, to the opinion leaders, and broadcast-quality public service announcements, in the form of DVDs, to the local TV channels. In the “only safe sap” area, the NGO trained *gachhis* on making *banas,* and encouraged using them on trees used for raw sap consumption. We also provided sweatshirts as an incentive to those *gachhis* who made and used *banas.*

Date palm sap is harvested during cold months from November to March [7]. We implemented a full “no raw sap” intervention from December 26, 2012 to March 29, 2013 in 342 villages in Rajbari District (Fig. 3). During the next sap harvesting season, from November 16, 2013 to January 31, 2014, we only broadcast the TV public service announcement. We implemented a full “only safe sap” intervention from October 3, 2013 to January 31, 2014 in 381 villages in Faridpur District, including a *gachhi* training component. We started the “only safe sap” intervention slightly before the sap season because we needed to train *gachhis* on making and using *banas* before they started collecting sap.

### Assessment of the intervention implementation

During the intervention implementation period, we received NGO weekly reports with photographs of the meetings. Our monitoring team visited 143 randomly selected villages to confirm placement of at least one poster, watched the TV public service announcements at least in one tea stall, and observed one meeting per village incognito. Tea stalls with a television set exist in almost every village, and serve as gathering places where men drink tea, watch television and chat with others. Since most of the villagers do not have television at home, this communication channel was used to target men. We also recruited 15 tea stalls with television access in each study area to monitor the number of times the TV public service announcement was broadcast daily. We collected written weekly reports from those tea stalls, indicating dates and times when the announcements were broadcast.

After the intervention, during April–May 2014, our quantitative data collection team interviewed 900 adult male and female respondents from 75 randomly selected villages from each “no raw sap” and “only safe sap” district. We described the sampling procedure for this study elsewhere [15]. Our data collection team asked about NiV knowledge, sap consumption behavior, use of *banas* and exposure to the interventions. In this manuscript, we only present data about the respondents’ direct exposure to the intervention.

### Assessment of the cost of developing and implementing the intervention

We used an activity-based costing approach to compare health interventions [16–20]. We identified, costed out, and quantified all development and implementation activities. We reviewed timelines and deliverables to confirm activities performed, transport requisition emails, and budgets submitted to the donor. We calculated the cost per activity performed using person time, with the exception of NGO activities that were calculated using per activity cost instead of person time cost.

We separated the start-up cost from the intervention implementation cost (Table 1). The start-up cost covered the development of materials before the implementation, from the period of time between the decision to implement, to the start of its delivery to the beneficiaries [21]. Because we developed some of the materials for both interventions, we were not able to completely separate the cost of developing all the materials for each intervention. Thus, we could not add the start-up cost to the implementation cost to determine the total cost per intervention.

**Table 1:** Description of activities and the cost of intervention development and implementation in Rajbari District 2012–2014 and in Faridpur District 2013–2014, to reduce the risk of Nipah virus infection in Bangladesh

Activities	Cost included
Intervention development /start-up cost	
Concept note and protocol development	Staff cost—international and local experts Cost of transportation Cost of materials development, testing and production Cost of venue for training the trainers
Explore communication channels	
District-selection field visit	
NGOs selection	
Communications organization selection	
Intervention materials development	
Materials testing	
TV campaign production	
Materials revisions	
Training of trainers	
Intervention implementation	
Production of print materials, stickers and sweatshirt	Production cost
Copies of TV materials	DVD cost
Training of NGO staff	Cost of training (venue, per diem, food and transportation of trainees). For *bana*-making training session; cost of bamboo, bana-making trainer
Opinion leaders and community meetings, placement of poster, *gachhi* meetings and incentive program	Cost of NGO field implementation
TV broadcasting	Cost of cable operators
TV monitoring	Cost of monitoring tea stalls
Intervention monitoring	Cost of the monitoring team

The implementation cost included NGO cost, mass media dissemination expenditures (local TV channel, DVDs copies and printing posters) and intervention monitoring cost. The cost of training NGO staff included training manuals, personnel, snack allowance, venue, electricity, photocopies, and transportation. In the “only safe sap” area, we also included the cost of *bana*-making materials and the allowance and transportation cost of a *bana*-making expert as part of the NGO staff training cost.

The cost of training the NGO staff and printing the materials would be incurred before any future implementation, thus we included them in the implementation cost.

We calculated the amount of money the NGOs spent as cost of the meetings and *gachhi* training. Since NGO staff affixed posters while visiting the villages for meeting purposes, the NGOs did not include the cost for placing posters separately in their reporting. To estimate this cost, we assumed that one person could visit four villages per day, to affix 10 posters per village, and estimated the cost of affixing one poster based on the daily salary, meal allowance and transportation costs. We deducted these costs from the meetings cost to calculate the cost per meeting. We calculated costs in US dollars, using a rate of 82.34 Bangladeshi takas per US$1, the conversion rate used on the original budget. We did not include the cost of the research study in this analysis.

### Data analysis

We calculated the start-up cost first, followed by the implementation cost of the interventions. We calculated cost per meeting by dividing the total cost to conduct all meetings, provided by the NGOs, by the total number of meetings conducted; and the cost per *gachhi* training by dividing the total training cost provided by the NGOs by total number of *gachhis* trained.

From our survey data, we calculated the percentage of people directly reached or exposed to each communication channel used during the intervention [22]. We found that a lower percentage of respondents from the “no raw sap” area reported that they were directly exposed to the intervention than the respondents from the “only safe sap” area (30% vs. 41%). Also a lower percentage of respondents reported exposure to each intervention component: TV public service announcement (11% vs. 12%), saw a poster (21% vs. 31%) and attend a meeting (10% vs. 12%) in the “no raw sap” area than the “only safe sap” area [22]. We calculated the cost per person reached per channel by dividing the implementation cost by the total population (361,000 in the “no raw sap” area and “335,000 in the “only safe sap” area) times the percentage of people reached per channel.

$${\text{Cost per person reached per component }} = \frac{\text{Total implementation cost}}{{{\text{Total population }} \times {\text{ Percentage of people reached}}}}$$

We estimated the future start-up cost and intervention implementation cost in all 30 districts where at least one NiV spillover has been identified in the past. We added person-day cost for activities, including the cost of revising the intervention and materials, identifying cable operators, cost for transportation and phone communication.

Using different risk-based scenarios in all 30 affected districts where 117 NiV spillovers were identified from 2001 to 2015 (unpublished NiV surveillance data), we estimated future implementation costs based on the number of spillovers per district. A spillover is defined as at least one identified NiV case in the district and we separated the districts into three categories:

- Six districts with six or more spillovers (48% of all spillovers)
- Thirteen districts with two to five spillovers (43% of all spillovers)
- Eleven district with one spillover, (9% of all spillovers).

We estimated the implementation cost at the district level, based on implementation expenditures during the 2012–2014 interventions.

To estimate the cost of the meetings for a future intervention, we estimated the number of rural households in all sub-districts using census data [23]. We projected conducting one opinion leaders meeting and one community meeting per every 500 rural households, using the cost-per-meeting from the “only safe sap” area. We projected the approximate number of *gachhis* using NGO data from the “only safe sap” area (3 *gachhis* per village or within 500 households). To estimate the cost of training the *gachhi*s we used the per-*gachhi* training cost from the “only safe sap” intervention. We assumed two cable operators per sub district to estimate the cost of broadcasting the TV public service announcement.

## Results

### Start-up cost of the 2012–2014 intervention

We incurred most of the start-up costs developing the intervention, including expenditures on national and international experts and local staff, materials’ pre-testing, revisions and production, districts and NGO selection, and training of trainers (Table 2). The second highest cost was the production of the TV public service announcements, followed by the cost of creating and producing the other communication materials.

**Table 2:** Start-up cost (preparation cost) for intervention development, materials development, production of materials, and training of trainers calculated using activity-based costing of an intervention to reduce the risk of Nipah virus infection conducted in two districts of Bangladesh in 2012–2013 and 2013–2014

Activities	Total (US$)
Intervention development	
Staff cost—international experts	$131,000
Staff cost—local experts	$36,050
Cost of creating the campaign and preparing materials for production	
Training manuals, TV public service announcements, posters, calendar, yearly planner, stickers, sweat shirts	$28,850
Materials pre-testing	
FGDs with local community. Materials were tested twice for the “no raw sap” intervention to get the Government approval. Materials were tested once for the “only safe sap” intervention	$1893
TV materials production^a^	
6-min docudrama and 3-min TV public service announcement for the “only safe sap” intervention	$39,940
Last minute revision of the intervention materials	
3-min TV public service announcement for the “no raw sap” intervention, new poster, revised calendar and revised training manuals	$9000
Training of trainers	
International expert trained 7 local experts to train NGO staff	$3342
Field visit for districts and NGO selection	
Transportation	$5716
Total	$255,791
Total in Bangladeshi taka (BDT)	21,061,831

*1US$* 82.34

^a^The TV public service announcement cost includes initial production and two revisions. The cost of both TV public service announcements are combined because the original shooting included footage for both versions of the TV public service announcements

### Intervention activities

The NGOs conducted 281 opinion leaders and 304 community meetings in the “no raw sap” area, and 381 opinion leaders and 220 community meetings in the “only safe sap” area. They affixed 3000 posters in the “no raw sap” area and 7000 posters in the “only safe sap” area. Local channels broadcast the TV public service announcements 5 times daily. In addition, in the “only safe sap” area, the local NGO conducted 1160 *gachhi* training sessions on how to make and use *banas*.

### Intervention implementation cost incurred during 2012–2014

Our implementation cost was lower in the “no raw sap” intervention than in the “only safe sap” intervention ($30,000 vs. $55,000) (Table 3). The cost of the intervention components, broadcasting the TV public service announcement ($313 vs. $674), promoting posters ($1305 vs. $2930) and conducting community meeting costs ($22,243 vs. $30,135) was lower in the “no raw sap” intervention than in the “only safe sap” intervention (Table 3).

**Table 3:** “No raw sap” and “only safe sap” intervention cost, implemented in 2012–2014 in Rajbari District and 2013–2014 in Faridpur District to reduce the risk of Nipah virus infection, Bangladesh

Component	“No raw sap” intervention (population: 361,000)			“Only safe sap” intervention (population: 335,000)		
	Description	Total cost (US$)	Cost per person reached (US$)	Description	Total cost (US$)	Cost per person reached (US$)
TV public service announcement						
Cable operator cost	1 operator in 2 sub districts at $154 each season for two seasons	$308		11 operators in 2 sub districts at $59.5 per operator for one season	$654	
DVDs cost	5 DVDs at $1 per unit	$5		20 DVDs at $1 per unit	$20	
Total cost of TV public service announcement	11% of people directly saw the TV public service announcement	$313	$0.008	12% of people directly saw the TV public service announcement	$674	$0.017
Poster						
Printing cost	3000 posters at $0.15 per unit	$450		7000 posters at $0.119 per unit	$830	
Affixing cost	3000 posters at $.30 per unit	$900		7000 posters at $.30 per unit	$2100	
Total cost of poster	21% of people saw a poster	$1305	$0.017	31% of people saw a poster	$2930	$0.028
NGO training						
Staff training	45 staff per training session, at $2674 per training session	$2674		56 staff per training session, at $2674 per training session	$2575	
Training manuals	1000 training manuals at $0.13 per unit	$125		1000 training manuals at $0.275 per unit	$275	
Total cost of NGO training		$2799			$2850	
Meeting cost						
Meetings	585 meetings at $37.48 per meeting	$21,928		601 meetings at $48.87 per meeting	$29,374	
Calendars/yearly planner	1500 yearly planners at $0.21 per unit	$315		5000 calendars at $0.152 per unit	$761	
Total cost of meetings	10% of people attended meeting	$22,243	$0.62	12% of people attended meeting	$30,135	$0.75
Intervention monitoring cost						
TV public service announcement monitoring	15 tea stalls per district at $1.3 per tea stall for two seasons	$40		15 tea stalls per district at $1.3 per tea stall for one seasons	$20	
Meeting monitoring	4 persons at $865per per person	$3460		4 persons at $865per per person	$3460	
Total cost of intervention monitoring		$3500			$3480	
Total cost with TV public service announcement, poster, meeting and monitoring of a “no raw sap” intervention	30% of people directly reached by the intervention	$30,205	$0.28			
*Gachhi* component						
Training				1160 *gachhis* at $7.6 per *gachhi* training	$8846	
Stickers to identify *bana* protected sap				6000 stickers, per $0.035	$210	
Incentive for gachhis who used *bana*				1100 *gachhis* at $5.8 per sweatshirt	$6346	
Total cost of *gachhi* component				1160 *gachhis* reached	$15,402	$13
Total cost with TV public service announcement, poster, meeting monitoring and *gachhi* training, for an “only safe sap” intervention				41% of people directly reached by the intervention	$55,471	$0.40
Total in Bangladeshi taka (BDT)		2,4487,080			4,567,564	

The cost per person directly reached by at least one intervention component was also lower in the “no raw sap” area than in the “only safe sap” area (28 cents vs. 40 cents).

The cost to reach one person per communication channel was lower in the “no raw sap” area than in the “only safe sap” area: TV public service announcement was 0.8 cents versus 1.7 cents, poster was 1.7 cents versus 2.8 cents, and community meetings was 62 cents versus 75 cents.

The cost of the *gachhi* training program in the “only safe sap” area, including the incentive of providing a sweatshirt to those observed using *banas* during follow up visits, was $15,000. The per *gachhi* cost with incentive was $13. With no incentive was $7.6 (Table 3).

### Estimated cost of scaling up to the NiV-affected region for a future intervention

To scale up the intervention, we estimated the start-up cost at $60,000 (Table 4; Additional file 1). Our future estimated implementation cost of meetings, posters and the public service announcement was the same for both the “no raw sap” and the “only safe sap” intervention (Table 5). However, the *gachhi* training component increased the cost of the “only safe sap” intervention. Thus, the implementation cost of a future intervention covering 30 districts would be $3.5 million using an “only safe sap” approach, and $2.6 million using a “no raw sap” approach (Table 5). The cost of printing and affixing the posters in 30 districts would be $96,000. Broadcasting the TV public service announcement in 30 districts would cost $26,000.

**Table 4:** Start-up cost to prepare a Nipah prevention intervention covering 30 Nipah-affected districts with at least one Nipah spillover, Bangladesh

Activities	Person	Person-days	Estimated cost
Developing intervention design	NiV and research and intervention expert (international)	10	$10,000
Contribute to intervention design and provide logistical support from the Government	NiV expert and intervention coordinator (from government)	10	Government contribution
Revising the intervention	Behavior change communication experts	20	$20,000
To write protocol and review NGO proposals	Assistant scientist	44	$4689
To identify and communicate with NGO and TV channel operators	Research officer^a^	154	$6303
	Field transportation^b^	140	$3401
Revising the intervention materials (poster, calendar, PSA, training guide)	Revision of the intervention materials (if needed)		$15,000
Cost of phone communication	(Communicating local NGO, local TV channels)		$448
Total			$59,841
Total in Bangladeshi taka (BDT)			4,927,308

^a^At $609 per month salary, at $15 per diem

^b^At 2000 taka (approximately $24) per day to rent a motorcycle to explore NGO and TV channels, about 4 days in one district and half a day inter-district travel

**Table 5:** Nipah prevention intervention implementation cost covering 30 Nipah-affected districts with at least one Nipah spillover, Bangladesh

Intervention element		NiV spillover 6 or more (total 56 spillover)	NiV spillover 2–5 (total 50 spillover)	NiV spillover 1 (total 11 spillover)	Nipah spillover all (total 117 spillover)
Component	Description	6 districts, consisting of 47 sub-districts with a rural population of 2,434,793	13 districts, consisting of 90 sub-districts with a rural population of 4,947,566	11 districts consisting of 76 sub-districts with a rural population of 4,276,269	30 districts with 213 sub-districts with a rural population of 11,658,628
		Cost (US$)	Cost (US$)	Cost (US$)	Total cost (US$)
TV public service announcement					
Cable operator cost	2 operators per sub district at $60 each	$5640	$10,800	$9120	
DVD	2 per cable operators at $1 per DVD	$188	$360	$304	
Total cost of TV public service announcement		$5828	$11,160	$9424	$26,412
Poster					
Printing cost	10 posters per every 500 households at $0.11 per unit	$5357	$10,885	$9408	$25,650
Affixing cost	10 posters per every 500 households at $.30 per unit	$14,610	$29,685	$25,659	$69,954
Total cost of poster		$19,967	$40,570	$35,067	$95,604
NGO training					
Staff training	Approximately 1 NGO to cover two sub-districts, 3 staff from one NGO to train a maximum of 50 staff per training session, at $2850 per training session	$5700	$8550	$8550	
Training manuals	60 training manuals per training session at $0.28 per unit	$34	$50	$50	
Total cost of NGO training		$5734	$8660	$8660	$23,054
Meeting cost					
Meetings	1 opinion leader and 1 community meeting per 500 households at $50 per meeting	$487,000	$989,500	$855,300	
Calendars	10 per opinion leader meeting at $0.15 per unit	$7305	$14,843	$12,830	
Total cost of meetings		$494,305	$1,004,343	$898,130	$2,396,778
Intervention monitoring cost					
TV public service announcement monitoring	10 tea stalls per district at $1.3 per tea stall	$611	$1170	$988	
Meeting monitoring	1 person in 1 district at $609 per month, at $15 (1200 taka) per diem, at $24 (2000 taka) transport per day, $1.2 (100 taka) per day phone bill	$9665	$20,942	$17,718	
Total cost of intervention monitoring		$10,276	$22,112	$18,706	$51,094
Total cost with TV public service announcement, poster, meeting and monitoring for a “no raw sap” intervention		$536,110	$1,086,845	$969,987	$2,592,942
*Gachhi* training^a^					
Training	3 *gachhis* per 500 households at $7.6 per *gachhi* training	$111,036	$225,606	$195,008	
Incentive	80% of *gachhis* at $5.8 per sweatshirt	$67,790	$137,738	$119,057	
Total cost of *gachhi* training		$178,826	$363,344	$314,065	$856,235
Total with TV public service announcement, poster, meeting monitoring and *gachhi* training for an “only safe sap” intervention		$714,936	$1,450,189	$1,284,052	$3,449,177
Total in Bangladeshi taka (BDT)		58,867,830	119,408,562	10,572,884	284,005,234

^a^We can get the cost of the “no raw sap” intervention excluding the cost of *“gachhi* training” component from the calculation

To implement an “only safe sap” intervention with community meetings, *gachhi* training, poster and the TV public service announcement in the six districts with 48% of all spillover would cost $715,000. To implement it in the second most affected area, thirteen districts with 43% of all spillover, would cost $1.5 million and in eleven districts with 9% of all spillover, would cost $1.3 million.

To implement a full “no raw sap” intervention with community meetings, posters and the TV public service announcement in the six most affected districts would cost $536,000. In the second most affected thirteen districts it would cost $1 million and another $970,000 to implement it in the other 11 districts.

## Discussion

We spent $30,205 implementing the “no raw sap” intervention and $55,471 on the “only safe sap” intervention. To scale these interventions up to 30 districts in Bangladesh where human infections with NiV have been identified, we estimated a cost of $2.6 million US$ for the “no raw sap” and $3.5 million US$ for the “only safe sap” intervention. NiV usually affects impoverished rural communities in Bangladesh, thus, affected families often experience a severe social and financial crisis [24, 25]. NiV kills people and leaves survivors with permanent neurological sequelae, similar to those experienced by some survivors of Japanese encephalitis [26, 27]. Sixty-one percent of NiV cases affected males with a mean age of 27 [3] who could be the main wage earners of the family. Most died [3], and those that survived could not continue to work due to the neurological effects of NiV. In addition, NiV is a disease that requires special care. Hospitalization and illness episodes can last a week [28]. The financial burden associated with hospitalization translates into reduced monthly food and children education expenditures, having to borrow money, taking loans with high interests, and selling assets [29–31]. Prevention could reduce the risk of disease transmission as well as save poor families from social degradation.

Despite the severity of Nipah illness, since an average of fewer than 20 NiV cases are identified annually in Bangladesh [3], the cost of NiV prevention is unlikely to meet the traditional criteria for cost-effective interventions to prevent cases [32]. However, in addition to causing sickness and death, outbreaks have social consequences including fear, social unrest, violence and economic loss [33–36]. For diseases with moderate to high perceived severity, such as pandemic influenza, SARS or Ebola, investing and intervening earlier in the outbreak can be cost effective [37]. NiV is a deadly disease that can transmit from person to person and represents a global pandemic threat [38, 39]. Estimating NiV prevention costs is of interest to local and global health communities, helping to make informed decisions on funding interventions to prevent this disease. If we prevent a large high-mortality NiV pandemic, an effective intervention would be remarkably cost-efficient.

Disaster preparedness reduces the impact of disasters and associated costs, compared to a scenario without preparedness [40]. Initiatives to mitigate low probability, high catastrophic risks are not uncommon. NASA spends millions of dollars each year to track asteroids, though chances of dying from an asteroid impact are very low for the average person in the United States [41]. Investing in active surveillance activities for zoonotic infections, implementing effective ecological health interventions, improving modeling capabilities, increasing evaluations of health systems and public health needs and policies, and implementing better risk communication can improve the preparedness to respond to emerging infectious diseases [42]. For example, Taiwan established a nationwide emergency department, based on a syndromic surveillance system, that collaborated with 189 hospitals for better public heath response to improve their pandemic flu preparedness and disease control capabilities [43]. Similarly, investing in preventing NiV could provide an important benefit.

Health intervention studies from Bangladesh, focusing on cost, find some similarities with our study [44–46]. A study on neonatal and child health reported a lower cost per person reached through local TV channels than other intervention components [46]. In our intervention, the cost of interpersonal communication was around 44 times higher than broadcasting the televised public service announcement in the “only safe sap” area. The estimated cost of posters was also low and could be integrated in future interventions.

Findings from our trial suggested more behavior change resulted from a one season “only safe sap” intervention than a two-season “no raw sap” intervention [22, 47]. This could be because the “only safe sap” intervention offered the option of drinking safe sap by promoting the use of *banas* among *gachhis,* an already existing behavior [7] that still allowed people to enjoy drinking sap. The *gachhi* training component might also have contributed to increased exposure to the intervention. Although its estimated scale up cost was higher than the “no raw sap” intervention, for upcoming seasons, the “only safe sap” intervention should be considered.

Spending US$ 3.5 million annually on an “only safe sap” intervention would be prohibitively costly for a low-middle income country like Bangladesh that currently spends only $30.83 per capita per year for healthcare [48] and 2.8% of gross domestic product in total health expenditures [49]. The high cost of the meetings used in this intervention makes it impossible to scale up and sustain this intervention without external funding. Reducing meetings and interpersonal communication would reduce costs and so increase the feasibility of scaling it up. We could achieve a lower cost intervention by including community health workers [50] and health workers from the Expanded Program of Immunization (EPI), as well as health workers from NGOs such as BRAC [51, 52]. They could conduct meetings in the areas immediately surrounding their offices, affix posters, provide leaflets, and disseminate messages to people receiving their services during the sap harvesting season, adding a minimal cost. In addition, eliminating the *gachhi* incentive for using *banas* would reduce the cost of the *gachhi* intervention by more than one-third.

Our intervention findings provide a framework to calculate costs of a future intervention to prevent NiV. However, the following limitations of our findings require consideration. We did not include the intervention impact data in the results of this cost manuscript, therefore, we cannot calculate cost-effectiveness. The complexity of the impact data required a separate manuscript to be properly presented. Nevertheless, this cost analysis, conducted from a provider’s perspective, enables future providers to weight the costs of taking on this intervention against those of other interventions [53]. Better understanding of the cost, from intervention providers and recipients, would provide an understanding of cost-related potential barriers and obstacles to implementing the intervention.

Although we calculated the separate cost of each intervention component, we cannot interpret the separate impact of each component. Since communication campaigns often rely on a synergistic effect, all of its components may need to run in parallel for maximum impact [54–56]. Therefore, although deploying only a single component markedly reduces cost, this body of work does not provide direct evidence that the standalone components will alter behavior.

To reduce costs, we proposed engaging government and other health workers to conduct meetings within their locality. Since, they already have other tasks to accomplish, small-scale pilot efforts could help identify practical strategies to integrate NiV prevention messages into health worker activities. The government already broadcast the “no raw sap” public service announcement during the 2015–2016 season. Continuing to measure the prevalence of raw sap consumption as these messages are disseminated more widely can provide useful guidance on adjusting interventions and messages going forward.

## Conclusions

Exploring low cost strategies to communicate prevention messages in frequently affected districts, such as broadcasting the public service announcement on local channels, combined with health workers visiting communities to spread messages and affix posters in districts with high risk of NiV spillover, may be an effective way to reduce the risk of NiV. Continuous monitoring efforts may help to further develop and refine the intervention components for more effective communication.

## Additional file

**Additional file 1.** Start-up cost to prepare the intervention covering 30 Nipah-affected districts with at least one Nipah spillover, Bangladesh, supporting document for Table 4.

## Abbreviations

NiV: Nipah virus infection
NGO: non-government organization
TV: television
DVD: digital video disk
